# High Prevalence of Autosomal Recessive Alport Syndrome in Roma Population of Eastern Slovakia

**DOI:** 10.3390/biomedicines13081960

**Published:** 2025-08-12

**Authors:** Gabriel Koľvek, Lucia Klimčáková, Gabriela Hrčková, Jozef Židzik, Ľudmila Podracká, Tatiana Baltesová, Kristína Kubejová, Jaroslav Rosenberger, László Barkai

**Affiliations:** 1 Department of Paediatrics and Adolescent Medicine, Faculty of Medicine, P. J. Šafárik University in Košice, 040 11 Košice, Slovakia; gabriel.kolvek@upjs.sk (G.K.); tatiana.baltesova@upjs.sk (T.B.); kristina.kubejova@upjs.sk (K.K.); laszlo.lajos.barkai@upjs.sk (L.B.); 2 Department of Medical Biology, Faculty of Medicine, P. J. Šafárik University in Košice, 040 11 Košice, Slovakia; jozef.zidzik@upjs.sk; 3 Department of Paediatrics, Faculty of Medicine, Comenius University in Bratislava, 833 40 Bratislava, Slovakia; gabriela.hrckova@nudch.eu (G.H.); ludmila.podracka@fmed.uniba.sk (Ľ.P.); 4 Transplant Department, Louis Pasteur University Hospital in Košice, 040 11 Košice, Slovakia; 5 Fresenius Medical Care—Dialyzačné Služby s.r.o., 040 11 Košice, Slovakia; jaroslav.rosenberger@upjs.sk; 6 2nd Department of Internal Medicine, Faculty of Medicine, P. J. Šafárik University in Košice, 040 11 Košice, Slovakia; 7 Olomouc University Social Health Institute, Palacký University Olomouc, 779 00 Olomouc, Czech Republic; 8 Physiological Controls Research Center, University Research and Innovation Center, Obuda University, 1034 Budapest, Hungary

**Keywords:** Alport syndrome, Roma population, autosomal recessive inheritance, founder effect, genetic screening

## Abstract

**Background/Objectives:** Alport syndrome (AS) predominantly presents with X-linked inheritance worldwide. However, the epidemiological landscape remains poorly characterized, particularly among ethnic minority groups like the Roma minority in Slovakia. Our study aimed to investigate the inheritance patterns of AS in this region and determine whether a distinct pattern predominates. **Methods:** Selective genetic screening for pathogenic variants previously occurring in Slovakia was performed. Samples from patients with persistent (familial) hematuria ± hearing loss who had not yet undergone biopsy or genetic testing were analyzed by high-resolution melting analysis. The prevalence of AS per million (pm) population was calculated by adding information on patients with previously confirmed AS. **Results:** Twenty-five new cases of ARAS, one digenic form, and two cases of XLAS were identified by screening. In total, we collected information on 46 patients with genetically or bioptically confirmed AS in the region of eastern Slovakia, corresponding to a prevalence of 29 pm population. The c.1598G>A (p.Gly533Asp) pathogenic variant of the collagen type IV alpha 4 chain, which follows an autosomal recessive inheritance pattern, was the most prevalent variant that was exclusively confirmed in Roma patients (*n* = 35), suggesting a founder effect. Within the Roma community, the prevalence of ARAS (the most prevalent inheritance pattern) corresponds to 133 pm of the Roma population, based on midpoint population estimates. **Conclusions:** Our findings demonstrate a unique genetic profile of AS in the Roma population, characterized by a high prevalence of ARAS, with implications for genetic counseling and screening strategies.

## 1. Introduction

Alport syndrome (AS) is a hereditary glomerular disease characterized by progressive kidney dysfunction, hearing loss, and ocular abnormalities [1]. The prevalence ranges from 1 in 5000 to 1 in 53,000 [2]. In the United States, AS accounts for approximately 2–3% of children and 0.2% of adults with ESRD [3,4], while in Europe it represents 0.6% of patients with ESRD [5]. X-linked inheritance, caused by mutations in the *COL4A5* gene, represents approximately 85% of cases in published cohorts, whereas autosomal recessive inheritance due to mutations in the *COL4A3* or *COL4A4* genes accounts for about 15% of cases [6,7,8].

Recent advances in genetic testing have revealed significant geographical and ethnic variations in inheritance patterns [9]. Increased prevalence of autosomal recessive Alport syndrome (ARAS) has only been found in few populations with identified founder mutations, including Brazil [10], Ashkenazi Jews [11], Cyprus [12], and Saudi Arabia [13]. Two founder variants contribute to the high prevalence of kidney failure in Czech Romani, with the estimated population frequency of ARAS due to these variants being at least 1:11,000 in Czech Romani [14].

The Roma population represents one of the largest minorities in Europe, widely dispersed [15], but most concentrated in Bulgaria, Hungary, Romania, Serbia, and Slovakia [16,17]. According to the 2021 census, only 67,179 persons were counted as Roma in Slovakia, representing approximately 1.23% of the population. However, this number is usually underreported, with estimates placing the actual Roma population at 7–11% of Slovakia’s total population, meaning there may be more than half a million Roma in the country [18]. The distinct genetic background and traditional endogamous practices of the Roma population [19] present a unique opportunity to study potential variations in inheritance patterns, including AS.

## 2. Materials and Methods

### 2.1. Study Population

This prospective study was conducted in eastern Slovakia (Košice and Prešov regions) from 1 January 2023 to 31 May 2025 to detect undiagnosed AS cases through selective genetic screening and to identify all confirmed cases for prevalence calculation.

### 2.2. Patient Identification and Recruitment

**Figure 1:**
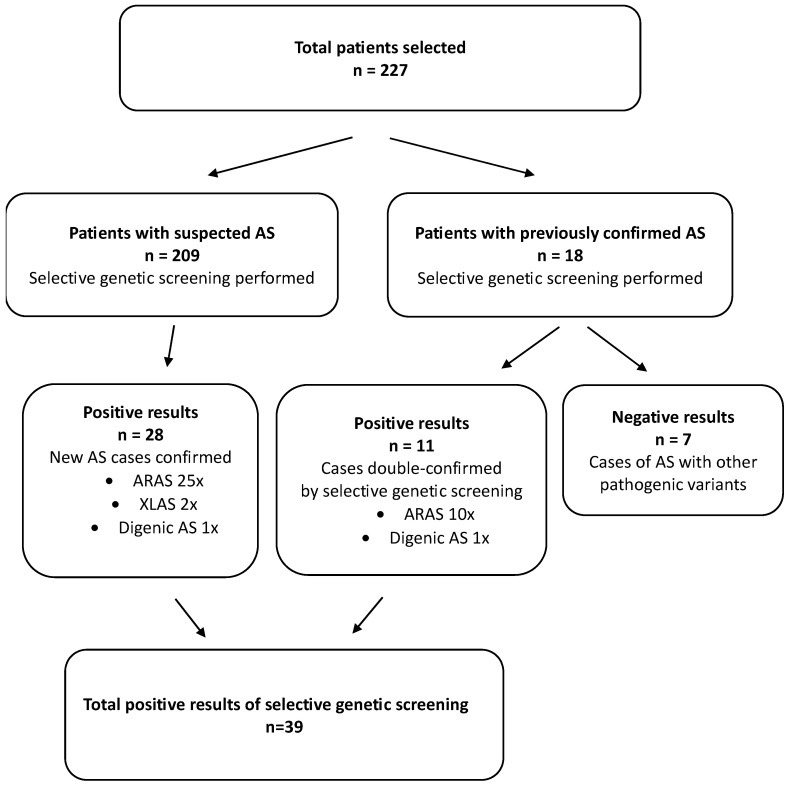
Patient selection and screening process.

Nephrologists were asked to identify and report patients with confirmed AS (biopsy-proven or with genetically confirmed pathogenic variants in the *COL4A3*, *COL4A4*, or *COL4A5* genes) as well as suspected cases of AS. Patients followed for (familial) hematuria with or without sensorineural hearing loss, and dialyzed or transplanted patients with chronic kidney disease (CKD) of unknown etiology were considered suspected of AS. We identified and investigated 227 patients: 209 candidates for selective genetic screening and 18 with previously confirmed AS diagnoses (Figure 1).

### 2.3. Genetic Analysis

Fifteen pathogenic variants of *COL4A3*, *COL4A4*, and *COL4A5* (see Supplementary Material) that we were aware of having occurred in Slovakia in the past in at least three unrelated patients were selected. Samples of EDTA blood for genetic testing were collected. All 15 variants of interest were analyzed by high-resolution melting analysis (HRMA) after real-time PCR using MeltDoctor™ HRM Master Mix (Applied Biosystems, Inc., Norwalk, CT, USA) with custom-designed primers and unlabeled probes on the Eco™ Real-Time PCR System (Illumina, Inc., San Diego, CA, USA). Genotypes were identified using Eco Real-Time PCR System Software v4.1.

HRMA was used due to lack of funds and relative unavailability of direct sequencing in Slovakia (impossibility of financial reimbursement through health insurance for a significant number of patients). It is a rapid, reliable, cost-effective closed-tube method and one of the most recent methods used in diagnostic genetic testing. The potential of HRM analysis for cost-effective and sensitive high-throughput genotyping and mutation scanning, as well as its comparison to other methods, has been reported in numerous studies and reviews [20,21,22].

The results were verified using positive samples (three positive samples previously identified by direct sequencing for each variant of interest were supplied from the Czech Republic and Bratislava, Slovakia). Two samples of each of the three mutations found in our patient group were also checked by direct sequencing, with 100% concordance.

The study was approved by ethical committees of the P. J. Šafárik University in Košice, L. Pasteur University Hospital in Košice, and Children’s Teaching Hospital in Košice, which are leading academic medical centers and teaching hospitals for adult and pediatric patients that serve as the primary referral centers enrolling patients from across the region. Written informed consent was obtained from adult patients (*n* = 120) and from parents or legal guardians of pediatric patients (*n* = 107).

### 2.4. Clinical Parameters

Normal growth of patients was defined as height between the 3rd and 97th percentile according to the data from the Public Health Authority of the Slovak Republic [23]. Normal final height was defined as 154.2 cm at minimum for a woman and 165.7 cm at minimum for a man (3rd percentiles). For the Roma population, no specific growth charts are available.

### 2.5. Statistics

The period prevalence of AS in eastern Slovakia was calculated using a three-step approach. First, overall prevalence was determined by dividing the total number of individuals with genetically or biopsy-proven AS diagnosed during the study period (January 2023—May 2025) by the average total population of eastern Slovakia (1,588,807), expressed per million inhabitants. Second, population-specific prevalence rates were calculated separately for Roma and non-Roma populations. For the Roma population, the number of AS cases among Roma individuals was divided by the estimated Roma population size in eastern Slovakia. Population denominators were based on the average population during the study period, with Roma population estimates derived from the Atlas of Roma Communities, while general population numbers were obtained from the Statistical Office of the Slovak Republic [18]. Third, prevalence rates between Roma and non-Roma populations were statistically compared using Fisher’s exact test, with relative risk (RR) and 95% confidence intervals calculated to quantify differences in AS prevalence between the two populations. Sensitivity analyses addressing uncertainty in Roma population estimates are provided in Supplementary Material. Statistical analyses were performed using IBM SPSS Statistics 25.0. The Mann–Whitney U test was used for non-parametric comparisons between groups. A *p*-value < 0.05 was considered statistically significant.

## 3. Results

Selective genetic screening was positive in 39 of 223 tested individuals (20 males). It revealed 28 previously unconfirmed patients with AS (25 ARAS, 2 XLAS, and 1 digenic) and re-confirmed previously biopsy-proven (*n* = 5) or genetically verified (*n* = 6) cases (Figure 1, Table 1).

**Figure 2:**
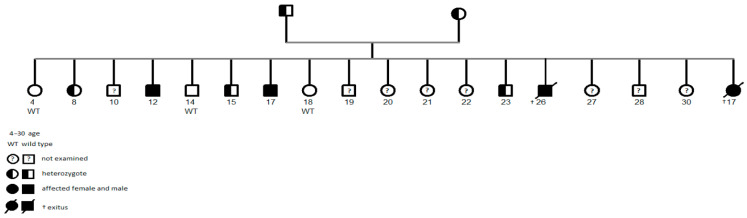
Pedigree of a Roma family with four ARAS-affected children.

Altogether, 35 patients (18 children) with an ARAS pathogenic variant c.1598G>A (p.Gly533Asp) of the alpha 4 chain from 25 families (1 family with 4 patients, 7 families with 2 patients) were identified at a median age of 17 years (IQR 11.5–31.5) in the region of eastern Slovakia, including five patients with previously biopsy-only confirmed AS (Table 1). All these ARAS patients were of Roma ethnicity. A pedigree of a Roma family with four children affected by ARAS is shown in Figure 2.

On top of 39 positively screened patients (including two XLAS cases), seven additional patients with previously diagnosed XLAS were referred but tested negative on screening. Three of these patients had previously been genetically confirmed to carry pathogenic variants other than those included in the screening panel, while four males had received their diagnosis based on kidney biopsy findings, positive family history, and hearing loss.

### 3.1. Epidemiology

We found the prevalence of AS in eastern Slovakia to be at least 29.0 pm in the general population. Considering the specific distribution of Roma within Slovakia—who are predominantly resident in the eastern part of the country (according to the Atlas of Roma Communities, approximately 16.6% of the region’s population, range: 13.2–20.0%)—the prevalence of AS is 140 pm (range: 116–176 pm depending on population estimates; ARAS 133 pm, range 110–167 pm). This contrasts sharply with the prevalence in the non-Roma population in the region, which was found to be 6.8 pm (range: 6.5–7.1 pm depending on population estimates), indicating that AS is 20.6 times more common in Roma (RR = 20.6; 95% CI 10.0–42.7; *p* < 0.001).

### 3.2. Presenting Symptoms of Patients with ARAS Pathogenic Variant c.1598G>A (p.Gly533Asp)

The most common presentation was incidental microscopic hematuria (*n* = 17). The median age (IQR) at identification of hematuria was 6 (5–10) years. Concurrent pathologic albuminuria at presentation occurred in nine cases (52.9%; median urine albumin-creatinine ratio 52 mg/mmol). Blood pressure was normal, and renal function corresponded to CKD stage 1 in all cases at presentation. No patient presented with hearing loss or visual disturbances. At the end of the study period, all patients remained in CKD stage 1.

Seven patients with the pathogenic ARAS variant c.1598G>A (p.Gly533Asp) presented with chronic nephritic syndrome accompanied by elevated serum creatinine (median 231 μmol/L; estimated glomerular filtration rate 31 mL/min/1.73 m^2^). The median age (IQR) at presentation was 16.5 (15–20) years. In six cases, AS was initially confirmed by kidney biopsy. In one case, diagnosis was confirmed by genetic testing alone without biopsy. Macroscopic hematuria was observed in two patients. All seven patients were prescribed nephroprotective medication; however, despite treatment, five patients progressed to kidney failure at a median (IQR) age of 25 (18–29) years. Of the remaining two patients, one currently has stage 4 chronic kidney disease, while the other maintains stage 2 with preserved renal function.

One patient aged 8 years presented with rapidly progressive glomerulonephritis (RPGN). Renal biopsy confirmed severe extracapillary glomerulonephritis with crescents, while electron microscopy demonstrated signs of a basket-weave pattern consistent with AS. Kidney function did not recover, and the patient died within 3 months of presentation due to malignant dysrhythmia.

Fourteen patients presented as dialysis crash-landers. Median age (IQR) at kidney failure was 23 (18.5–27.5) years, which was not significantly different from the age at kidney failure in the subgroup presenting with chronic nephritic syndrome (*p* = 0.80).

### 3.3. Growth and Associated Features of Pathogenic Variant c.1598G>A (p.Gly533Asp)

Except for one child with a history of intrauterine growth restriction (IUGR), all patients with ARAS in CKD stage 1 (*n* = 14) demonstrated normal growth patterns. Among the adult cohort, two men had heights below 165.7 cm, and one woman had a height below 154.2 cm. These patients had progressed to CKD stages 3–5 by the time they reached adulthood.

Hearing loss was documented in 22 ARAS patients at a median age of 20 years (IQR 17–26). Three patients demonstrated normal audiometry results at a median age of 4 years (IQR 4–10). Audiometry was not performed in 10 children (median age 9.5 years, IQR 7–12.5) who lacked clinical signs of hearing loss, including three cases where testing was recommended but not completed due to non-adherence.

## 4. Discussion

Our study demonstrates a marked deviation from typically reported inheritance patterns of AS, particularly within the largest ethnic minority in Europe, the Roma population. Specifically, we observed a high prevalence of the c.1598G>A (p.Gly533Asp) pathogenic variant among Roma individuals in eastern Slovakia. This predominance strongly suggests a founder effect within this population, a phenomenon well-documented in genetically isolated communities [24] and is consistent with documented historical migration patterns and the community’s endogamous marriage practices [15,25].

### 4.1. Epidemiology

Our findings of a significantly increased AS prevalence in the Roma population of eastern Slovakia (140 pm, representing a 20.6-fold increased risk compared to the non-Roma) align remarkably well with recent genetic findings from the neighboring Czech Republic reported by Plevova et al. [14]. Their study identified two founder pathogenic variants—c.1598G>A (p.Gly533Asp) in *COL4A4* and c.415G>C (p.Gly139Arg) in *COL4A3*—that account for most ARAS cases in Czech Romani, with an estimated prevalence of at least 1:11,000 in this population. Notably, Romani patients represented 76% of all ARAS cases in their cohort despite comprising only 2.8% of the Czech population, which is consistent with our epidemiological findings in Slovakia. The authors noted that consanguinity in Romani communities likely contributes to the high prevalence of these recessive variants.

### 4.2. Clinical Presentation and Progression of Patients with ARAS Pathogenic Variant c.1598G>A (p.Gly533Asp)

The results of our selective genetic screening indicate that the disease typically manifests during early childhood in both genders, presenting with microscopic hematuria [26]. Importantly, this hematuria is usually detected incidentally during routine check-ups or through targeted examinations in patients with a positive family history, which is consistent with published literature [27,28,29].

The clinical outcomes in our cohort are consistent with the key findings of Gross et al. [30], who demonstrated that treatment of advanced AS cases with established proteinuria and chronic kidney disease is much less effective than early intervention. Our findings follow this pattern, as shown by fourteen dialysis “crash-landers” who reached kidney failure at a median age of 23 years, and, more notably, the seven patients with the pathogenic ARAS variant c.1598G>A (p.Gly533Asp) who, despite receiving timely nephroprotective therapy after presenting with chronic nephritic syndrome, still progressed to kidney failure in 71% of cases (5/7 patients) at a median age of 25 years.

Early genetic diagnosis [31] and potential novel therapies [32] may improve future outcomes. Recent reviews show promise for advanced genetic technologies, including CRISPR gene editing and viral vector-mediated gene therapy approaches [33,34]. Additionally, improved understanding of genotype-phenotype correlations is enabling earlier patient identification. These diagnostic and therapeutic advances offer new hope for patients with AS, as genetic testing becomes increasingly available and research efforts continue to expand [35]. Our findings add to the growing literature on genotype-phenotype correlations in AS [36,37,38,39]. However, since most of our patients with AS were recently identified through screening and have limited follow-up data, our current observations become increasingly valuable as these patients are monitored over time.

### 4.3. Screening Strategy Implications

The high prevalence of ARAS observed in our cohort suggests that diagnostic and screening protocols for AS may benefit from population-specific considerations. This observation is particularly relevant given the extensive evidence demonstrating that distinct inheritance patterns significantly influence disease progression patterns, with autosomal recessive patients exhibiting more severe disease progression [40,41]. We therefore propose implementing targeted screening protocols specifically for the Roma population. Given the remarkably high frequency of the c.1598G>A (p.Gly533Asp) pathogenic variant in this community, such an approach would optimize case detection efficiency while reducing per-case screening expenditures, resulting in significantly improved cost-effectiveness ratios relative to population-wide screening initiatives.

At the same time, our findings highlight an important ethical tension between screening efficiency and healthcare equity. While targeted genetic testing proves cost-effective for populations with founder effects, it may inadvertently perpetuate health disparities by systematically underdiagnosing disease in genetically diverse populations. Future screening programs must therefore balance population-specific approaches with equitable access to genetic diagnosis across all communities.

### 4.4. Strengths and Limitations

Several limitations should be considered when interpreting our findings. Our use of targeted genetic screening for 15 specific mutations likely introduced significant ascertainment bias that limits the validity of cross-population prevalence comparisons. While this approach effectively captured cases in the Roma population due to a suspected founder effect with one predominant mutation, it systematically underdiagnosed cases in the majority population, where greater genetic diversity means most pathogenic variants fall outside our testing panel. This methodological bias likely exaggerated the prevalence difference between populations. The Roma population appeared to have higher disease rates primarily because our genetic test was more effective at detecting their common mutation, not necessarily because they actually have more disease. Furthermore, our limited geographical scope and potential referral bias may limit the broader applicability of our findings.

Despite these limitations, our study provides important contributions to the field. This represents the first systematic investigation of AS in eastern Slovakia, providing valuable baseline data for future research. Our targeted approach was well-suited for detecting the predominant mutation in the Roma population, and our findings highlight important population-specific differences that warrant further investigation. Future studies involving comprehensive sequencing approaches and larger, more diverse populations are needed to obtain unbiased prevalence estimates and validate our findings.

### 4.5. Future Directions and Recommendations

Future research should focus on creating genetic screening programs specifically for the Roma population to identify Alport syndrome cases early. Extended longitudinal studies are needed to validate these initial findings and establish comprehensive genotype-phenotype correlations across diverse Roma populations. Additionally, genetic counseling programs should be developed that are tailored to the Roma community’s specific cultural background, ensuring that families receive clear, understandable information about the condition and inheritance patterns. These efforts will improve early detection, patient care, and family planning decisions for this at-risk population.

## 5. Conclusions

Our results demonstrate a remarkably high prevalence of ARAS in the Roma population, Europe’s largest ethnic minority, with a substantially elevated risk compared to the majority population in Slovakia. Our findings may inform clinical practice and public health strategies across European regions with significant Roma populations by highlighting the critical need for targeted screening and genetic counseling programs. These data support the implementation of preventive measures in affected communities.

## Figures and Tables

**Table 1:** Patients screened positive.

Inheritance Pattern	COL4 Chain	Pathogenic Variant	Age at Verification	CKD Stage at Verification	Previous Verification	Ethnicity
ARAS	Alpha 4	c.1598G>A (p.Gly533Asp)	12	1	-	R
ARAS	Alpha 4	c.1598G>A (p.Gly533Asp)	11	1	-	R
ARAS	Alpha 4	c.1598G>A (p.Gly533Asp)	5	1	-	R
ARAS	Alpha 4	c.1598G>A (p.Gly533Asp)	5	1	-	R
ARAS	Alpha 4	c.1598G>A (p.Gly533Asp)	10	1	-	R
ARAS	Alpha 4	c.1598G>A (p.Gly533Asp)	8	1	-	R
ARAS	Alpha 4	c.1598G>A (p.Gly533Asp)	17	1	-	R
ARAS	Alpha 4	c.1598G>A (p.Gly533Asp)	4	1	-	R
ARAS	Alpha 4	c.1598G>A (p.Gly533Asp)	24	5	-	R
ARAS	Alpha 4	c.1598G>A (p.Gly533Asp)	15	1	-	R
ARAS	Alpha 4	c.1598G>A (p.Gly533Asp)	26	5	-	R
ARAS	Alpha 4	c.1598G>A (p.Gly533Asp)	20	5	-	R
ARAS	Alpha 4	c.1598G>A (p.Gly533Asp)	36	5	-	R
ARAS	Alpha 4	c.1598G>A (p.Gly533Asp)	50	5	-	R
ARAS	Alpha 4	c.1598G>A (p.Gly533Asp)	11	4	-	R
ARAS	Alpha 4	c.1598G>A (p.Gly533Asp)	39	5	-	R
ARAS	Alpha 4	c.1598G>A (p.Gly533Asp)	35	5	-	R
ARAS	Alpha 4	c.1598G>A (p.Gly533Asp)	27	5	-	R
ARAS	Alpha 4	c.1598G>A (p.Gly533Asp)	21	5	-	R
ARAS	Alpha 4	c.1598G>A (p.Gly533Asp)	n.a.	5	-	R
ARAS	Alpha 4	c.1598G>A (p.Gly533Asp)	17	5	-	R
ARAS	Alpha 4	c.1598G>A (p.Gly533Asp)	n.a.	1	-	R
ARAS	Alpha 4	c.1598G>A (p.Gly533Asp)	15	1	-	R
ARAS	Alpha 4	c.1598G>A (p.Gly533Asp)	28	5	-	R
ARAS	Alpha 4	c.1598G>A (p.Gly533Asp)	32	5	-	R
ARAS	Alpha 4	c.1598G>A (p.Gly533Asp)	38 (15)	5 (3)	KBx	R
ARAS	Alpha 4	c.1598G>A (p.Gly533Asp)	35 (20)	5 (3)	KBx	R
ARAS	Alpha 4	c.1598G>A (p.Gly533Asp)	25 (21)	5 (3)	KBx	R
ARAS	Alpha 4	c.1598G>A (p.Gly533Asp)	31 (18)	5 (3)	KBx	R
ARAS	Alpha 4	c.1598G>A (p.Gly533Asp)	8 (8)	5 (5)	KBx	R
ARAS	Alpha 4	c.1598G>A (p.Gly533Asp)	17 (15, 16)	4 (3, 4)	G + KBx	R
ARAS	Alpha 4	c.1598G>A (p.Gly533Asp)	20 (12, 19)	1 (1, 1)	G + KBx	R
ARAS	Alpha 4	c.1598G>A (p.Gly533Asp)	16 (15)	5 (5)	G	R
ARAS	Alpha 4	c.1598G>A (p.Gly533Asp)	4 (3)	1 (1)	G	R
ARAS	Alpha 4	c.1598G>A (p.Gly533Asp)	16 (15)	1 (1)	G	R
Digenic	Alpha3 + Alpha4	c.1598G>A (p.Gly533Asp) c.415G>C (p.Gly139Arg)	13 (11)	1 (1)	G	R
Digenic	Alpha3 + Alpha4	c.1598G>A (p.Gly533Asp) c.415G>C (p.Gly139Arg)	40	5	-	R
XLAS	Alpha 5	c.1871G>A (p.Gly624Asp)	48	n.a.	-	Non-R
XLAS	Alpha 5	c.1871G>A (p.Gly624Asp)	17	1	-	Non-R

KBx = kidney biopsy-proven diagnostics, G = genetic-proven diagnostics, n.a.: not available; the age at verification with kidney biopsy or genetics is written in parentheses; the CKD stage at verification with kidney biopsy or genetics is written in parentheses; R: Roma, non-R: non-Roma.

## Data Availability

Data presented in this study is contained within the article and Supplementary Material. Further inquiries can be directed to the corresponding author.

## Supplementary Materials

The following supporting information can be downloaded at https://www.mdpi.com/article/10.3390/biomedicines13081960/s1: Supplementary File S1: List of pathogenic variants tested selectively by screening; Supplementary File S2: Sensitivity Analysis: Roma population estimates and AS prevalence.
