# Supplementary material for: High Prevalence of Autosomal Recessive Alport Syndrome in Roma Population of Eastern Slovakia
Source: Biomedicines. 2025 Aug 12;13(8):1960. doi: 10.3390/biomedicines13081960 (PMC12383956; doi:10.3390/biomedicines13081960)
Supplement: Supplementary file 1 [file biomedicines-13-01960-s001.zip › Supplementary File S2_Roma population estimate.pdf]

Supplementary File S2. Sensitivity Analysis: Roma population estimates and AS prevalence

	**Roma population**	**Total population**	**AS cases in Roma**	**AS cases per million Roma population**	**ARAS cases per million Roma population**
**Minimal population estimate**	210,040 (13.2%)	1,588,807	37 (35)	176	167
**Medium population estimate**	263,979 (16.6%)	1,588,807	37 (35)	140	133
**Maximal population estimate**	317,917 (20.0%)	1,588,807	37 (35)	116	110

The second column presents estimate number (%) of Roma of the total population of eastern Slovakia (i.e. population of Kosice and Presov region, which is shown in the third column). The fourth column shows the number of all Alport syndrome (AS) cases, with autosomal recessive AS (ARAS) cases shown in parentheses.
