# Supplementary material for: High Prevalence of Autosomal Recessive Alport Syndrome in Roma Population of Eastern Slovakia
Source: Biomedicines. 2025 Aug 12;13(8):1960. doi: 10.3390/biomedicines13081960 (PMC12383956; doi:10.3390/biomedicines13081960)
Supplement: Supplementary file 1 [file biomedicines-13-01960-s001.zip › Supplementary File S1 - List of path variants.pdf]

Supplementary File S1:

List of pathogenic variants tested selectively by screening

COL4A3 c.1594 G>T (p.Gly532Cys)

COL4A3 c.415G>C (p.Gly139Arg)

COL4A4 c.1598G>A (p.Gly533Asp)

COL4A4 c.3707G>A (p.Gly1236Glu)

COL4A4 c.1045 C>TA (p.Arg349*)

COL4A4 c.1033dupG (p.Asp345Glyfs*85)

COL4A4 c.1933G>A (p.Gly645Arg)

COL4A4 c.4421C>T (p.Thr1474Met)

COL4A4 c.2164G>A (p.Gly722Ser)

COL4A4 c.1716 delT (p.Pro573Leufs*80)

COL4A4 c.2833G>A (p.Gly945Arg)

COL4A5 c.1871G>A (p.Gly624Asp)

COL4A5 c.3508G>A (p.Gly1170Ser)

COL4A5 c.1032+3_1032+6 delAAGT

COL4A5 c.3942G>C (p.Gln1314His)
